# Long-term combined organic and inorganic fertilization enhances soil quality and crop yield by regulating bacterial diversity and soil physicochemical properties

**DOI:** 10.3389/fmicb.2026.1909648

**Published:** 2026-08-03

**Authors:** Wenbo Mi, Jianjun Zhang, Shuying Wang, Tinglu Fan, Gang Zhao, Yi Dang, Lei Wang, Gang Zhou, Xujiao Zhou, Jingyu Hu, Shangzhong Li

**Affiliations:** 1 Institute of Dryland, Gansu Academy of Agricultural Sciences, Lanzhou, China; 2 Key Laboratory of Low Carbon Green Agriculture in Northwestern China, Ministry of Agriculture and Rural Affairs, Lanzhou, China; 3 Key Laboratory of Higher Water Utilization on Dryland of Gansu Province, Lanzhou, China

**Keywords:** bacterial community, bacterial diversity, organic fertilizer, soil properties, soil quality index

## Abstract

**Introduction:**

Soil microorganisms are widely recognized as a key indicator of soil quality. However, how the application of organic fertilizers drives soil quality and crop yield through microbial pathways is still unclear.

**Methods:**

A long-term field experiment conducted on the Loess Plateau evaluated the effects of different fertilization treatments on soil properties, microbial communities, and crop yields. The treatment groups included no fertilization (CK), inorganic nitrogen (N), inorganic phosphorus (P), organic fertilizer alone (M), nitrogen-phosphorus compound fertilizer (NP), and a combination of organic fertilizer and nitrogen-phosphorus compound fertilizer (MNP).

**Results:**

Both MNP and M treatments significantly increased the soil quality index (SQI) and grain yield, with the MNP treatment showing a more pronounced effect than the M treatment. SOC and TN were identified as the main factors influencing SQI. Compared with CK, the MNP treatment increased wheat and maize yields by 208.04 and 103.19%, respectively. Under the MNP treatment, the relative abundance of dominant bacterial phyla and genera increased, among which the increases in *Pseudomonadota* and *Chthoniobacter* were significantly correlated with soil properties. Furthermore, the MNP treatment also led to an increase in bacterial alpha diversity. Regression analysis indicated that bacterial alpha diversity was positively correlated with the contents of SOC, TN, MBN, MBC, and NH₄^+^-N, but negatively correlated with soil pH. Both bacterial community composition and diversity positively contributed positively to SQI. The main factors driving the increase in bacterial alpha diversity included pH, WFPS, NO₃^−^-N, Olsen-P, and NH₄^+^-N.

**Discussion:**

In conclusion, the long-term combined application of organic and inorganic fertilizers can improve soil quality, enhance bacterial diversity, and maintain crop yields.

## 1 Introduction

The widespread use of chemical fertilizers has significantly increased crop yields in the short term, but long-term excessive application disrupts the composition and diversity of soil microbial communities (Islam et al., 2022). As key drivers of nutrient cycling, microorganisms are responsible for organic matter transformation and nutrient mineralization (Qi et al., 2025; Zhou et al., 2024). When microbial functions are inhibited, the soil’s nutrient supply capacity declines, ultimately leading to reduced crop yields (Wan et al., 2024; Yang et al., 2022). Therefore, maintaining a healthy microbial community is key to reducing chemical fertilizer use while achieving high and stable crop yields.

Manure is a cornerstone of sustainable agriculture, as it alleviates long-term challenges such as soil degradation and the decline in microbial diversity caused by the excessive application of chemical fertilizers (Mustafa et al., 2020; Sun et al., 2025). The incorporation of manure into cropping systems is widely advocated to improve soil fertility and crop resilience (Lyu et al., 2025a). Soil microorganisms govern the carbon and nitrogen cycles, processes that are closely associated with organic matter decomposition. Manure application facilitates the proliferation of microbial communities in agricultural soils (Gao et al., 2021). Furthermore, manure addition enhances fungal community diversity in both bulk and rhizosphere soils (Hontoria et al., 2019). Recently, it has been reported that the combined application of straw and manure strengthens the mutualistic symbiosis between arbuscular mycorrhizal fungi and crop roots, thereby increasing nutrient absorption. Additionally, it has been well established that the application of manure increases soil organic carbon stocks and enhances enzyme activity (Hinsinger et al., 2014; Liu et al., 2024). Nevertheless, Systematic studies on the effects of bacterial and fungal community features on soil quality under long-term manure application remain scarce.

Multiple factors regulate soil microbial composition and diversity, such as soil texture, pH, nutrient availability, and organic matter content (Linton et al., 2020). Manure, as a main source of soil organic matter, directly adds exogenous organic carbon and nutrients, thus affecting the structure and function of microbial communities (Sharma et al., 2016). Manure application improves soil aggregate structure, reduces bulk density, and increases total nitrogen (TN) and organic carbon (SOC) content. These improvements create favorable habitats for microbial proliferation and activity (Mder and Berner, 2012). Regarding biological properties, manure application has been shown to significantly increase soil microbial biomass carbon (MBC) and nitrogen (MBN), as well as the activities of extracellular enzymes such as urease, phosphatase, and dehydrogenase (Liu et al., 2024). Furthermore, pH is a crucial environmental factor highly sensitive to microbial activity. Manure application buffers soil pH, alleviating acidification or alkalization stress, thereby making the microbial community more diverse and stable (Giannetta et al., 2018; Luo et al., 2016). In short, manure not only provides a direct carbon source for microorganisms but also indirectly creates a more favorable growth environment by improving the soil’s physical and chemical properties, thereby influencing the microbial community.

Microbial metabolites are a key component of the soil’s stable carbon pool (Wang et al., 2021). Microorganisms break down plant debris and utilize the carbon it contains to obtain the energy they need for growth. Similarly, applied manure mixes with the soil and is decomposed by microorganisms, thereby accelerating microbial metabolism (Raven and Wagner, 2021; Wu et al., 2024). Consequently, we hypothesize that long-term application of manure improves soil quality and increases crop yields by enhancing the abundance and diversity of beneficial microbial communities in the soil. However, existing research on manure application is largely limited to specific application modes and lacks a comprehensive assessment based on multiple factors. Moreover, the mechanisms by which manure regulates microbial communities to affect soil quality remain unclear. This study aims to quantify soil quality under different fertilizer management strategies, analyze the correlations between soil bacterial and fungal communities, dominant microbial taxa, and soil properties following long-term application of manure, and elucidate the effects of microbial community composition and diversity on soil quality. Ultimately, the study seeks to reveal the potential mechanisms by which manure application improves soil quality through microbial regulation.

## 2 Materials and methods

### 2.1 Experimental site

The long-term tillage and fertilization experiment was established in 2005 at the Northwest Dryland Nutrition and Fertilization Science Observation and Experiment Station of the Ministry of Agriculture and Rural Affairs, located in Wutong Village, Shangxiao Town, Zhenyuan County, Qingyang City, Gansu Province, on the Loess Plateau of eastern Gansu (35°29′42″N, 107°29′36″E; altitude 1,279 m). The region is a typical rain-fed agricultural area. Over the past 30 years, the mean annual precipitation has been 510 mm, with over 60% occurring from July to September, showing high interannual and seasonal variability. The annual potential evaporation is 1,532 mm and the frost-free period lasts 170 days. The initial soil chemical properties in the 0–30 cm layer were as follows: organic matter 14.46 g kg^−1^, total nitrogen 1.11 g kg^−1^, total phosphorus 0.77 g kg^−1^, total potassium 28.22 g kg^−1^, alkali-hydrolyzable nitrogen 78.7 mg kg^−1^, available phosphorus 13.9 mg kg^−1^, and available potassium 150 mg kg^−1^.

### 2.2 Experimental design and management

The experiment employed a randomized complete block design and included the following six treatments: no fertilizer (CK), mineral nitrogen (N 150 kg ha^−1^, N), mineral phosphorus (P₂O₅ 105 kg ha^−1^, P), manure-only (decomposed pure cattle manure 22,500 kg ha^−1^ on a dry weight basis, M), mineral nitrogen and phosphorus (N 150 kg ha^−1^ + P₂O₅ 105 kg ha^−1^, NP), and combined mineral fertilizer and manure (N 150 kg ha^−1^ + P₂O₅ 105 kg ha^−1^ + manure fertilizer 22,500 kg ha^−1^ on a dry weight basis, MNP). The nutrient content of the manure (on a dry weight basis) was as follows: organic matter 15.2%, nitrogen 0.43%, phosphorus 0.45%, and potassium 0.28%. Each plot area was 72 m^2^ (8 m × 9 m), with three replicates. The basal-to-topdressing ratio for nitrogen fertilizer was 5:5. For winter wheat, topdressing was manually broadcast after rainfall during the greening stage. For spring maize, topdressing was manually applied in holes at the jointing stage. Farmyard manure and phosphorus fertilizer were applied once as basal fertilizer before sowing. Fertilizers were incorporated into the soil during pre-sowing rotary tillage. The crop rotation followed a sequence of one year of spring maize followed by 3 years of winter wheat. To minimize experimental error due to varietal differences, a uniform winter wheat cultivar., ‘Longjian 301’, was used with a seeding rate of 157.5 kg ha^−1^, sown manually in furrows with a row spacing of 20 cm. The spring maize cultivar was ‘Shendan 16’, planted at a density of 5.0 × 10^4^ plants ha^−1^, with a row spacing of 50 cm and plant spacing of 40 cm. Both cultivars are characterized by strong stress resistance and high yield potential. The winter wheat stubble height was maintained at approximately 3–5 cm, with roots allowed to decompose naturally in the field, while the remaining aboveground straw was completely removed. The maize stubble height was maintained at approximately 5–8 cm. During tillage, both maize and wheat stubble were incorporated into the soil. All other management practices followed conventional requirements. The manuscript presents data from one crop rotation cycle covering the period of 2022–2025 (Spring maize was planted from April to September 2022, and winter wheat was planted from September 2022 to July 2025, Supplementary Figure S1).

### 2.3 Sampling and measurement

#### 2.3.1 Collection and analysis of soil samples

In July 2022 and September of 2023–2025, during the harvest periods of spring maize and winter wheat, soil samples were collected from the 0–30 cm layer (stratified at 10 cm intervals). A five-point sampling method was used. After mixing, divide the sample into two portions. One portion was immediately preserved with dry ice and quickly sent to the microbiology laboratory, while the other portion was air-dried to determine its physicochemical properties. SOC was measured using the K₂Cr₂O₇–H₂SO₄ oxidation method (Walkley, 1935). TN was analyzed using an elemental analyzer. The pH was determined using a pH meter with a soil-to-water ratio of 1: 2.5. NH₄^+^-N and NO₃^−^-N contents were measured using a flow injection analyzer. MBN and MBC were quantified using the chloroform fumigation-extraction method (Vance et al., 1987). In addition, soil BD was determined using the core method. The calculation of WFPS was performed according to the established protocol for determining soil pore water saturation (Gomes et al., 2009). Additionally, the content of water-stabilized soil aggregates was determined using the wet sieve method.

#### 2.3.2 Assessment of soil quality

For the measured indicators (including pH, SOC, TN, NH₄^+^-N, NO_3_^−^-N, Olsen-P, MBC, MBN, WFPS, BD, and soil aggregate), the total data set (TDS) method was used to evaluate the soil quality index (SQI) following the standard approach for soil quality assessment (Doran and Parkin, 2015). Principal component analysis is used to determine the proportion of variance in the common factor explained by each factor, which is then used as the weight for each indicator. A nonlinear scoring function was applied to standardize and assign scores to the soil physicochemical indicators, as follows (Shukla et al., 2006):


$$\mathrm{FNL}=\frac{x}{1+{\left(x/x_{i}\right)}^{m}}$$

The nonlinear transformation score for each indicator is denoted as FNL, with an actual value of x and a mean of xᵢ. Indicator scores are set on a two-way scale: m = −2.5 represents “the more, the better,” and m = 2.5 represents “the less, the better.” The formula for calculating the SQI is as follows (Andrews et al., 2002): $\mathrm{SQI}=\sum_{i=1}^{n}W_{i}\times \mathrm{FN}L_{i}$ where *FNL_i_* represents the score of indicator *i*; *n* represents the number of indicators, and Wi represents the weight of indicator *i*.

#### 2.3.3 Grain yield

The spring maize and winter wheat were harvested separately from each plot when the crops reached maturity. Then, it was air-dried, threshed, and weighed. The grain exhibited a moisture content of 14% (maize) and 13% (wheat) at the time of threshing.

#### 2.3.4 Sequencing of bacterial and fungal communities in the soil

After extracting DNA from the soil samples, the V3 + V4 region of 16S rDNA was amplified using barcoded, species-specific primers. The primer sequences were: 341F: CCTACGGGNGGCWGCAG; 806R: GGACTACHVGGGTATCTAAT. Amplify the ITS2 region of the ITS using barcoded, species-specific primers. The primer sequences for ITS were: ITS3_KYO2: GATGAAGAACGYAGYRAA; ITS4: TCCTCCGCTTATTGATATGC. To construct a sequencing library, the purified amplification products were ligated with sequencing adapters, and then subjected to on-board sequencing using the Illumina platform.

After raw reads were obtained from sequencing, they were first filtered and corrected using DADA2, and non-redundant reads along with their corresponding abundance information were output. The reads were then merged into tags, followed by the removal of chimeric tags to obtain the tag sequences and abundances for subsequent analysis, i.e., ASV sequences and ASV abundance information. The ASV sequences and abundance data served to annotate species, analyze composition and indicators, assess alpha/beta diversity, and predict community functions (Chao, 1984; Shannon, 1948; Simpson, 1949). Microbial sequencing was completed by Guangzhou Aozhi Biotechnology Co., Ltd., and the raw data of the sequenced microorganisms have been deposited in the NCBI SRA under accession number PRJNA1465133.

### 2.4 Statistical analysis of data

Data normality and homogeneity of variance were tested using the mean values obtained from the three soil layers. One-way analysis of variance (ANOVA) followed by the least significant difference (LSD) test (*p* < 0.05) was used to explore variations in soil properties, SQI, and maize yield. Mantel tests were conducted in RStudio using the corrplot, tidyverse, and ggplot2 packages, while structural equation modeling (SEM) was carried out with the piecewise SEM package. All figures were generated using Origin 2021 and R 4.4.2.^1^

## 3 Results

### 3.1 Soil properties

**Figure 1:**
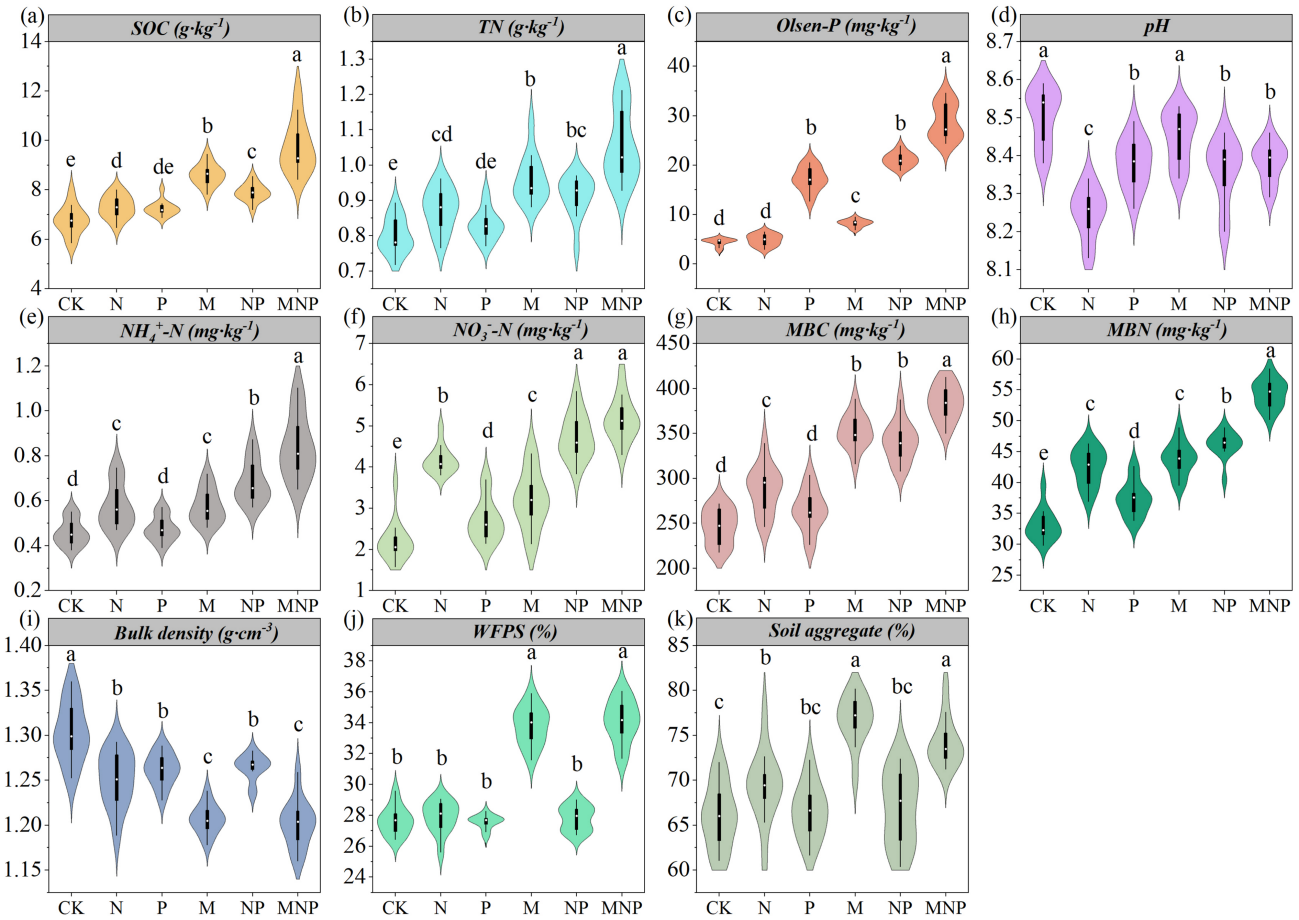
Soil properties under different fertilization patterns. Each treatment contains 12 sample data covering the years 2022–2025. Significant differences among treatments are indicated in different lowercase letters. **(a)** SOC. **(b)** TN. **(c)** Olsen-P. **(d)** pH. **(e)** NH_4^+^_-N. **(f)** NO_3^-^_-N. **(g)** MBC. **(h)** MBN. **(i)** Bulk density. **(j)** WFPS. **(k)** Soil aggregate.

Compared with chemical fertilizer treatments (N, P, and NP), MNP significantly increased the levels of SOC, TN, NH₄^+^-N, Olsen-P, MBC, MBN, WFPS, and soil aggregate (Figures 1a–c,e,g,h), while it decreased pH (Figure 1d). The SOC, TN, Olsen-P, NH₄^+^-N, MBC, MBN, WFPS, and soil aggregate levels were higher under MNP than NP, with increases of 23.70, 16.48, 36.49, 23.53, 12.89, and 22.15%, respectively.

Compared with NP, N, P, and CK, manure fertilizer treatments (M and MNP) significantly enhanced WFPS and soil aggregate content (Figures 1j,k), while reducing soil bulk density (Figure 1i). Specifically, MNP decreased BD by 7.2% and increased WFPS by 12.5% relative to NP. In addition, compared with NP, MNP resulted in a 4.76% decrease in BD and 22.62 and 11.79% increases in WFPS and soil aggregate, respectively.

### 3.2 Soil quality and maize yield

**Figure 2:**
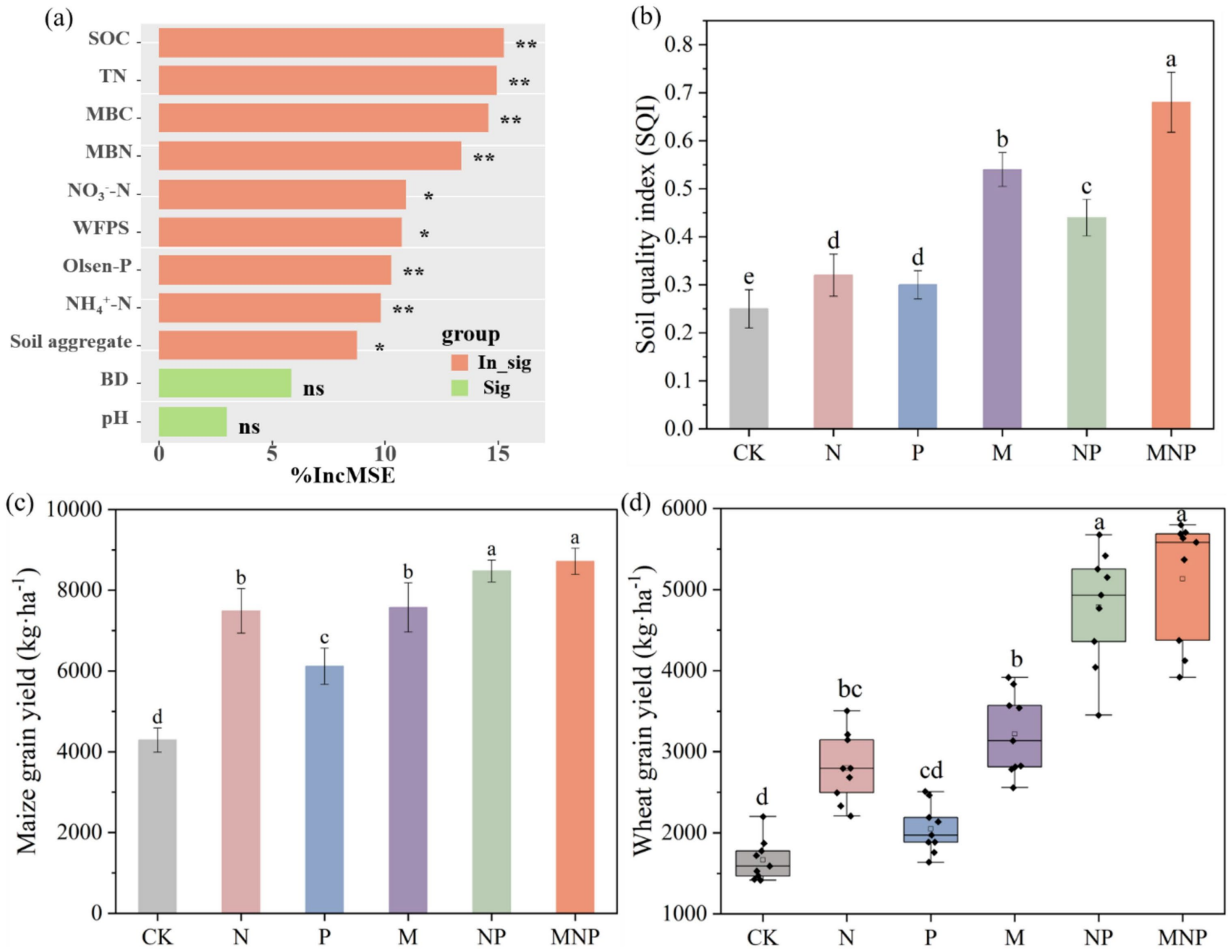
Soil quality index, crop yields, and the contributions of soil factors to SQI under different fertilization management strategies: **(a)** Contributions of soil factors to the SQI **(a)**, SQI **(b)**, spring maize yield (2022) **(c)**, and winter wheat yield (2022–2025) **(d)**. Significant differences between treatments are indicated by different lowercase letters. *p < 0.05; **p < 0.01; ns, *p* > 0.05.

Random forest analysis indicated that soil properties including SOC, TN, MBC, and MBN contributed most significantly to the SQI (Figure 2a). Under various fertilization management treatments, the ranking of SQI values was as follows: MNP > M > NP > N > P > CK (Figure 2b). Specifically, the SQI values under the MNP and M treatments were 54.54 and 22.73% higher, respectively, than those under the NP treatment. Compared with the NP treatment, the SQI values under the N, P, and CK treatments were 37.50, 46.67, and 76.00% lower, respectively.

The rankings of wheat and maize yields under different fertilization management practices were as follows: MNP > NP > M > N > P > CK. Compared with CK, the maize yield under MNP and M treatments increased by 103.19 and 76.57%, respectively (Figure 2c). Furthermore, between 2022 and 2025, the wheat yield under MNP and M treatments increased by 208.04 and 93.22%, respectively, compared with CK (Figure 2d). There was no statistically significant difference in wheat and maize yields between the NP and MNP treatments (*p* > 0.05).

### 3.3 Characteristics of the soil microbial community

#### 3.3.1 Diversity and composition of soil microbial community

**Figure 3:**
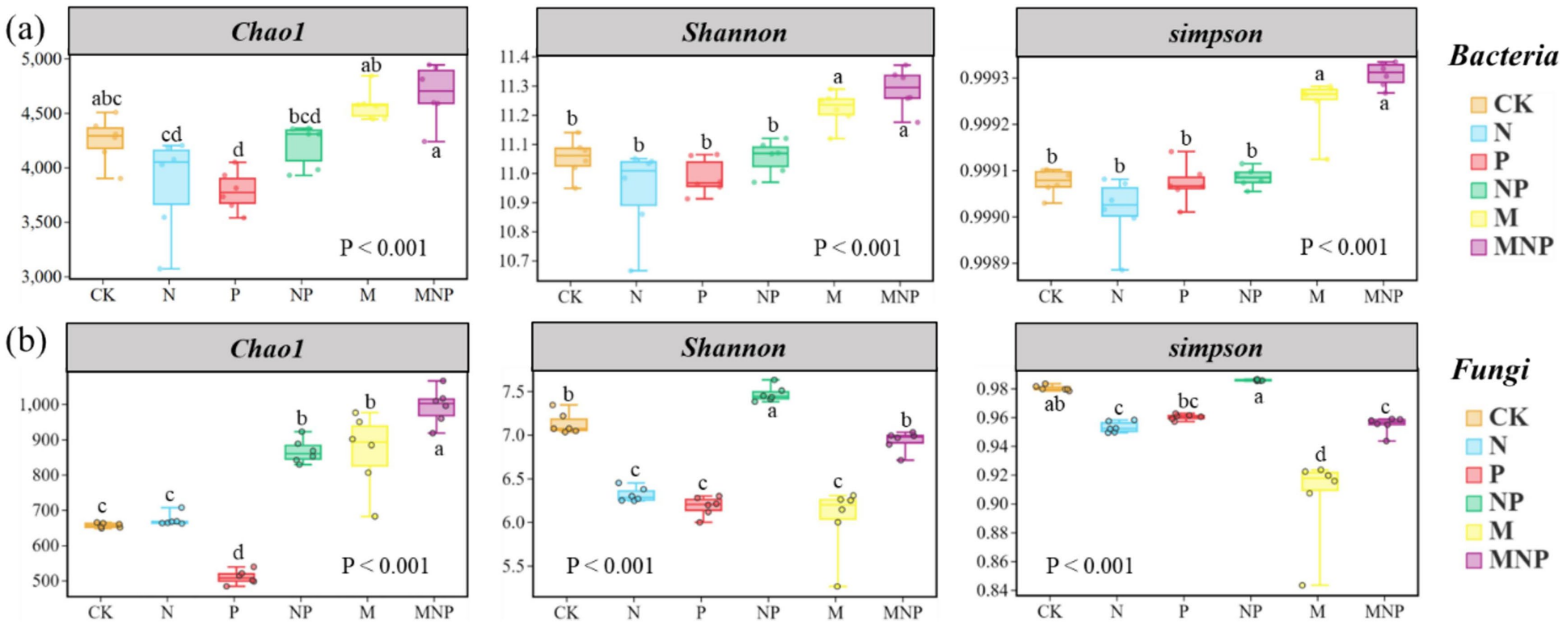
Alpha diversity of soil bacterial and fungal communities under different fertilizer management strategies. **(a)** Soil bacterial diversity. **(b)** Soil fungal diversity. Chao1, Shannon, and Simpson index are used to represent the alpha diversity of microbial communities. Significant differences among treatments are indicated in different lowercase letters.

Compared with CK and NP, the MNP and M treatments significantly increased soil bacterial diversity (*p* < 0.05), with no significant (*p* > 0.05) difference observed between the MNP and M treatments (Figure 3a). Furthermore, compared with CK, the MNP and M treatments increased the soil fungal Chao1 index while decreased the Shannon and Simpson indices (Figure 3b).

**Figure 4:**
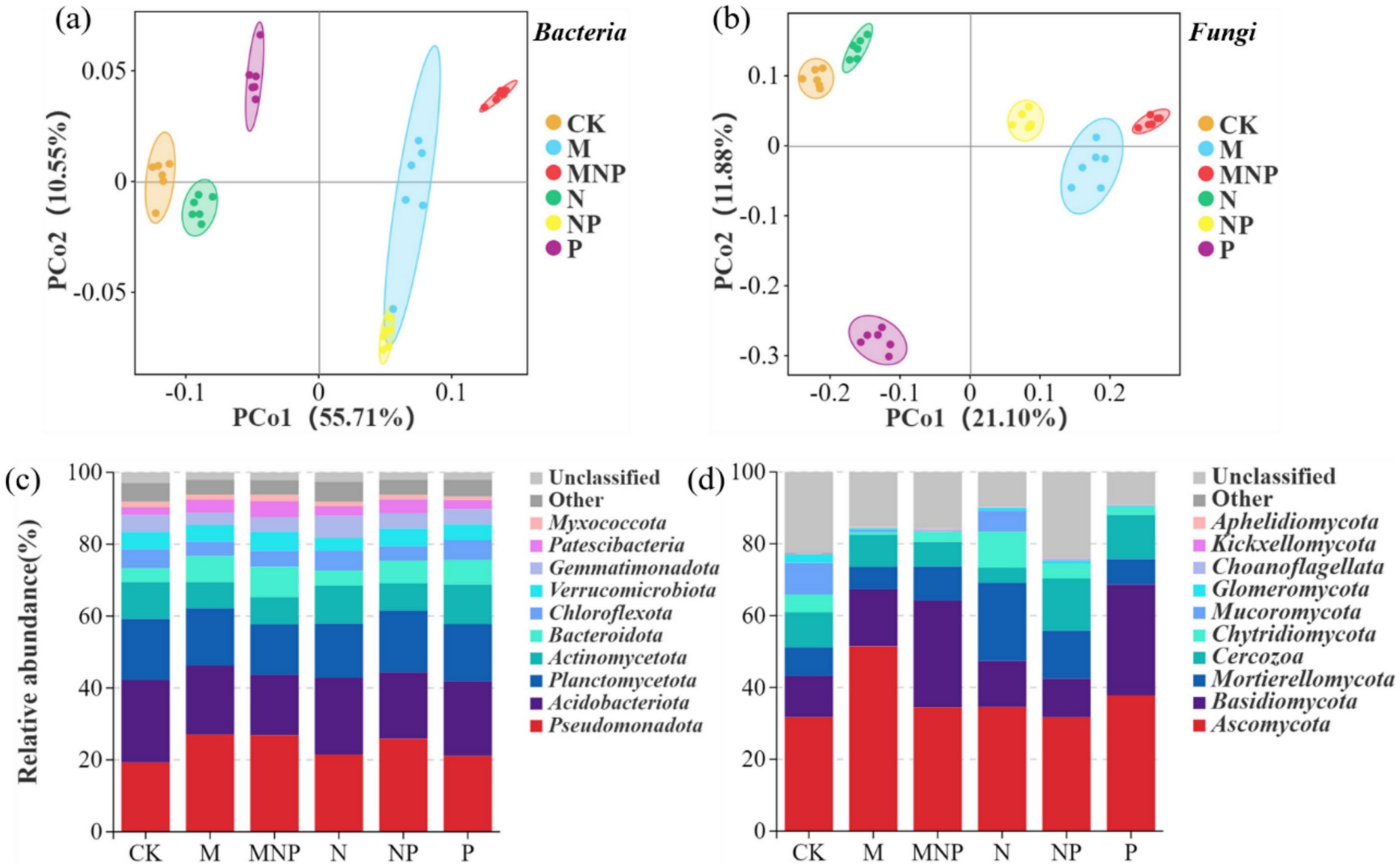
PCoA and phylum-level composition of soil bacterial and fungal communities under different fertilization management strategies. **(a)** PCoA of soil bacteria. **(b)** PCoA of soil fungi. **(c)** Soil bacterial composition. **(d)** Soil fungal composition.

According to the PCoA analysis, different treatments induced significant changes in microbial community composition (*p* < 0.05). PCo1 and PCo2 explained 55.71 and 10.55% of the bacterial community variation, respectively (Figure 4a). Clear differences were observed among treatments. Bacterial communities were similar under M and NP treatments. However, for the fungal community, the results showed low similarity among different treatments (Figure 4b). Similar results were also obtained from the PCA analysis (Supplementary Figure S2).

In the soil bacterial community, *Pseudomonadota*, *Acidobacteriota*, *Planctomycetota*, *Actinomycetota*, and *Bacteroidota* were identified as the dominant phyla (Figure 4c). Compared with CK, the relative abundances of *Pseudomonadota* under the MNP and M treatments increased by 39.08 and 42.11%, respectively. The relative abundance of *Acidobacteriota* under the CK treatment was higher than that under the MNP and M treatments, by 35.80 and 19.16%, respectively. At the genus level, similar patterns were observed in the bacterial communities across treatments. The dominant genera under the MNP and M treatments were *Sphingomonas* and *Lysobacter*, whereas MND1 was dominant under the CK and N treatments (Supplementary Figure S3a).

The predominant fungal phyla in the soil included *Basidiomycota*, *Ascomycota*, *Mortierellomycota*, *Cercozoa*, and *Chytridiomycota*, collectively accounting for over 60% of the total fungal community (Figure 4d). Compared with the other treatments, the M treatment resulted in a higher relative abundance of *Ascomycota*. Under the MNP treatment, the relative abundance of *Basidiomycota* increased by 158.28 and 175.77%, respectively, compared with CK and NP. At the genus level, *Alternaria* and *Leucoagaricus* exhibited higher abundances under the M treatment than under the other treatments, whereas *Lepista* showed the highest abundance under the MNP treatment (Supplementary Figure S3b).

#### 3.3.2 Relationships between soil property and microbial communities

**Figure 5:**
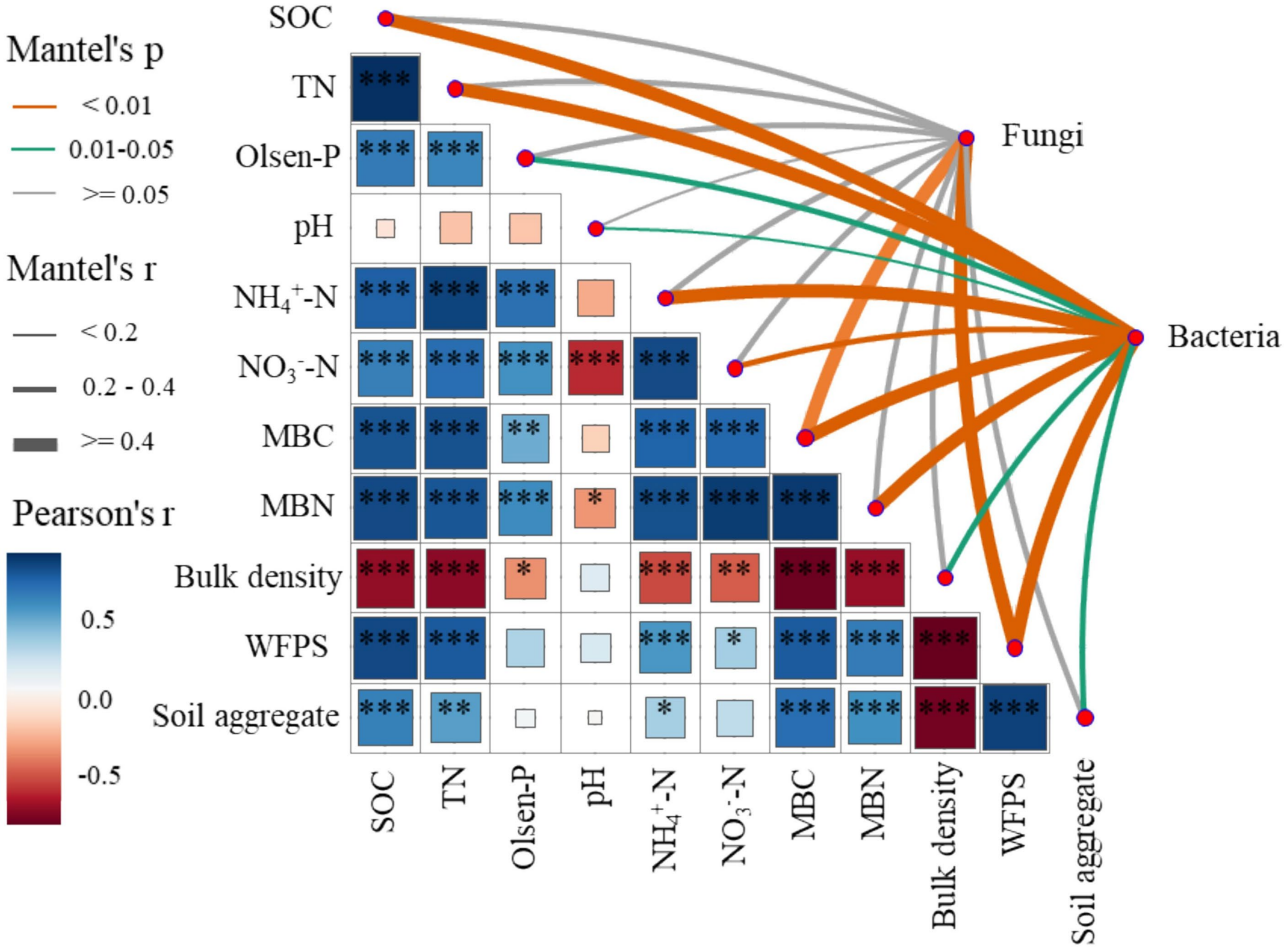
Mantel test showing soil factor correlations with soil bacterial and fungal communities. The width and thinness of the lines indicate the strength of the relationship between soil microbial community and soil factors. The color of the squares indicates the correlation between soil factors. *p < 0.05; **p < 0.01; ***p < 0.001.

According to Mantel tests, environmental factors exerted a significant influence on bacterial community composition (*p* < 0.05), but showed no significant correlation with the most fungal community composition (*p* > 0.05; Figure 5). Among the environmental factors themselves, strong correlations were detected. BD was negatively correlated with all other factors except pH, with significance at *p* < 0.05 or *p* < 0.01 levels. pH was negatively associated with most factors. With the exception of pH and BD, all remaining factors exhibited significant (*p* < 0.05 or *p* < 0.01) positive correlations with each other.

The cumulative variation in the bacterial community at the genus level was 74.64% for RDA1 and 13.90% for RDA2 (Supplementary Figure S4a). The dominant bacteria in the MNP treatment were negatively correlated with BD and pH, while positively correlated with the other factors. *Chthoniobacter* (belonging to *Verrucomicrobia*), *Flavisolibacter* (belonging to *Bacteroidota*), and *Ramlibacter* (belonging to *Pseudomonadota*) exhibited the most significant (*p* < 0.05) correlations with environmental factors and were also closely associated with biological characteristics such as MBN and MBC.

**Figure 6:**
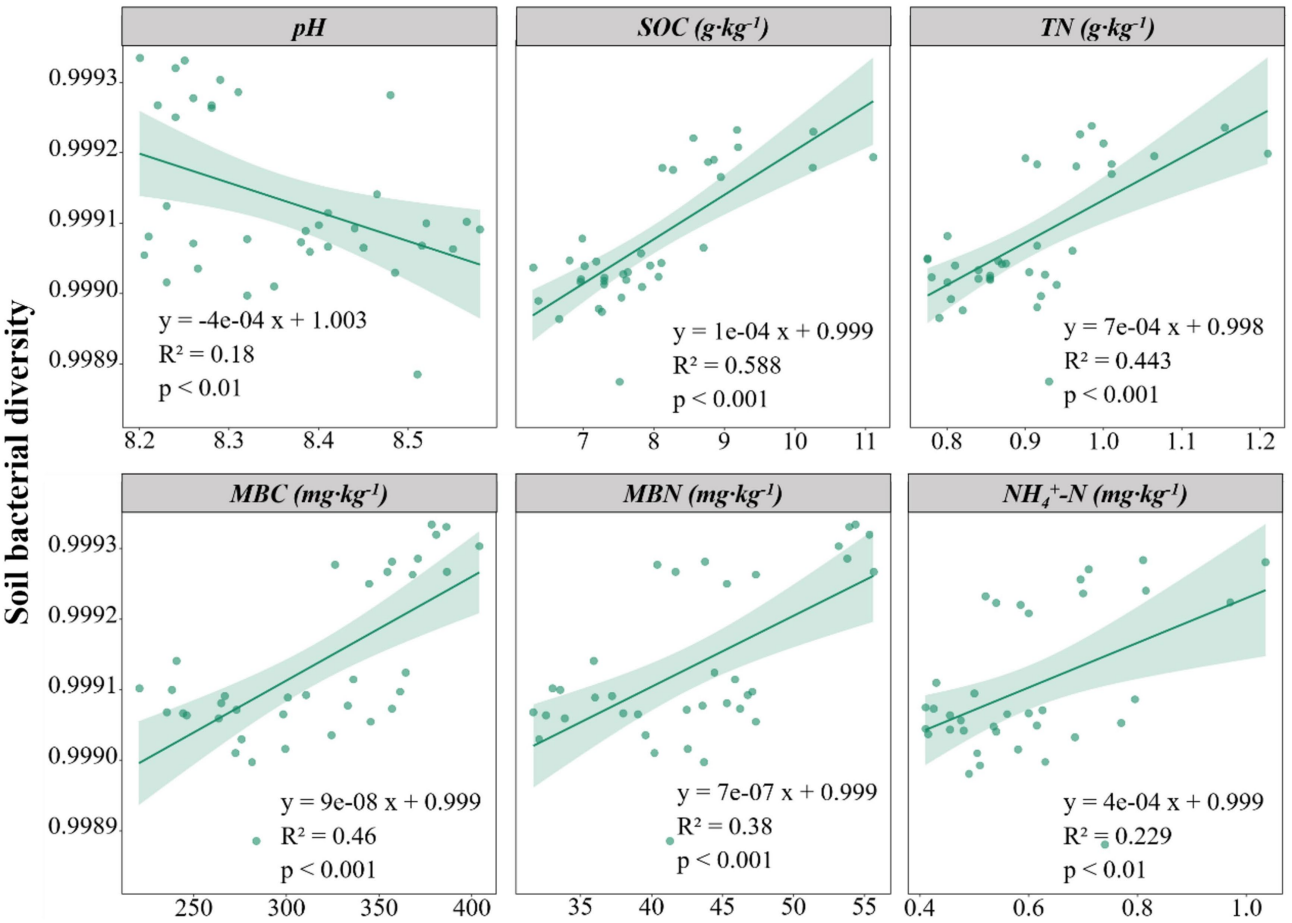
Correlation between soil bacterial communities and soil factors. Bacterial diversity was assessed using the Simpson index.

The cumulative variation in the fungal community was 48.98% for RDA1 and 28.31% for RDA2 (Supplementary Figure S4b). Therefore, manure addition primarily influenced the soil bacterial community by altering soil environmental factors. Regression analysis indicated that soil bacterial diversity was positively correlated with SOC, TN, NH₄^+^-N, MBC, and MBN, while negatively correlated with soil pH. (Figure 6).

### 3.4 Overall impacts of soil environmental factors and bacterial community attributes on SQI

**Figure 7:**
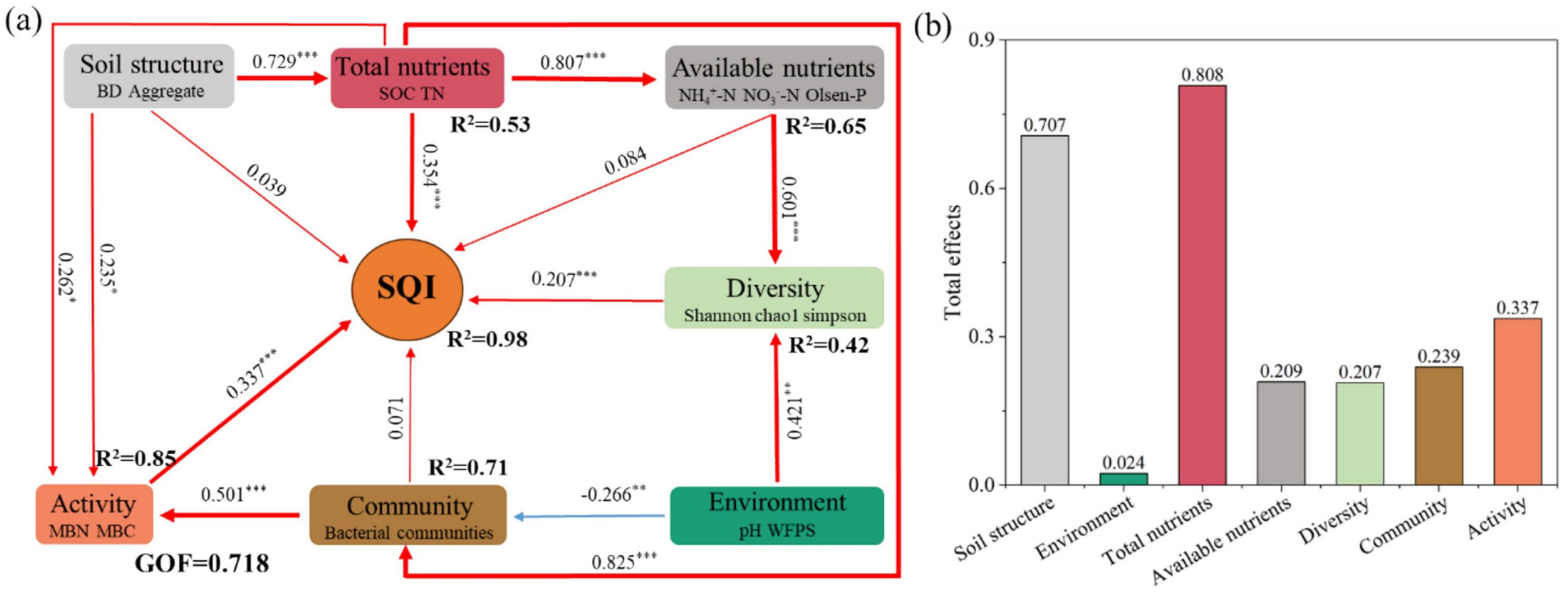
Pathways showing how the different soil properties impact the soil quality index **(a)**. Blue and red arrows represent significant negative and positive relationships, respectively (*p < 0.05; **p < 0.01; ***p < 0.001). Given the strong correlation between factors within the various variables, the environment, available nutrients, total nutrients and microbial activity, diversity, and community factors were downscaled by a principal component analysis before PLS-PM and the correlation analysis. A bar chart visualizes total effects based on PLS-PM **(b)**.

Direct positive effects on SQI were revealed by the structural equation model, which examined the interrelationships among soil environmental factors, bacterial diversity, bacterial community composition, and SQI. These effects were attributed to soil physical structure (BD, aggregate), soil nutrients (SOM, TN, NO₃^−^-N, NH₄^+^-N, Olsen-P), microbial activity (MBN, MBC), bacterial community composition, and diversity (Figure 7a). Bacterial diversity was positively influenced by soil environment (SPC = 0.421**) and available nutrients (SPC = 0.601***). Meanwhile, total soil nutrients positively affected SQI indirectly via microbial activity (SPC = 0.262*) and bacterial community composition (SPC = 0.825***). These pathways showed that the increase in bacterial diversity was driven by changes in pH, NO₃^−^-N, Olsen-P, and NH₄^+^-N. Figure 7b present the total effects of each soil environmental factor, as well as microbial community and diversity, on SQI.

## 4 Discussion

### 4.1 Manure improves soil quality by raising soil nutrient levels

Consistent with previous studies on organic amendments, this study demonstrates that, while ensuring food production, manure addition improves soil physicochemical and biological properties (Tian et al., 2022). The increases in SOC, TN, and available nutrients (Olsen-P and NH₄^+^-N) under the MNP treatment align with the established view that exogenous organic material serves as a direct carbon and nutrient source (Figure 1), thereby enhancing soil fertility (Ed-Haun et al., 2007). A long-term experiment on black soil found that replacing a portion of chemical fertilizers with manure increased SOC and TN levels by 58.7 and 66.4%, respectively (Zhang et al., 2025). The decrease in soil pH observed after manure application is attributed to the buffering capacity of organic matter, which reduces acidification stress and establishes a more favorable environment for microbial activity (Guo et al., 2010). Notably, manure application significantly improves soil physical properties, as evidenced by increased WFPS and aggregate content and reduced bulk density. These improvements are consistent with findings that organic amendments promote soil aggregation and structural stability. Similar findings have been reported in studies involving green manure, in which the application of organic matter similarly improved soil structure and enhanced soil aggregates (Lyu et al., 2025b; Yin et al., 2025). The enhancement of soil physical structure likely facilitates better water retention and aeration, thereby indirectly promoting microbial proliferation and nutrient cycling. The substantial increases in MBC and MBN under the MNP treatment indicate that manure addition stimulates microbial growth and activity. This finding suggests that manure accelerates microbial metabolism and promotes the formation of a more active microbial community (Kong et al., 2011). Furthermore, SOC, TN, MBC, and MBN were identified as the key contributors to the SQI, underscoring the pivotal role of microbial biomass and soil organic matter in governing soil quality (Figure 2). In conclusion, long-term manure application, particularly when combined with chemical fertilizers, effectively enhances soil quality by improving physicochemical properties, increasing microbial biomass, and optimizing soil physical structure, thereby promoting sustainable agricultural production.

### 4.2 Manure application increases dominant bacterial abundance and bacterial community diversity

The addition of organic materials affects soil microbial community composition and diversity by changing soil properties (Hu et al., 2026). Under manure application, *Pseudomonadota* became the dominant bacterial phylum (Figure 4c). *Pseudomonadota* are a dominant group widely found in organic-rich soils (Thonar et al., 2017). Their metabolic diversity enables them to secrete various extracellular enzymes, such as proteases, phosphatases, and dehydrogenases, thereby effectively decomposing complex organic compounds (Breitkreuz et al., 2020). Furthermore, the synergistic interaction between manure application and microorganisms including *Pseudomonadota* promotes the mineralization of organic nitrogen and organic phosphorus (Pereira and Castro, 2014). This process leads to the generation of numerous small-molecular organic acids, siderophores, and phytohormones, which play crucial roles in activating soil nutrients, suppressing soil-borne pathogens, and promoting crop root growth (Thonar et al., 2017). This interaction optimizes the rhizosphere microecological environment, providing crops with higher nutrient availability and enhanced stress resistance (Shen et al., 2001). Notably, at the genus level, dominant bacteria were substantially more abundant under the MNP treatment than under the M treatment (Figure S3).

A significant influence of environmental factors was detected on bacterial community composition (*p* < 0.05), but not on fungal community composition, which showed no significant correlation with most environmental factors (*p* > 0.05) (Figure 5). This is consistent with previous findings indicating that bacterial communities are more sensitive to changes in soil environment than fungal communities (Kong et al., 2011; Yang et al., 2019). Research shows that bacteria are more efficient than fungi at using simple organic compounds, whereas fungi are better at using complex ones. The structural equation model indicated that soil bacterial communities are positively influenced by total nutrient levels, particularly SOC and TN. It has been previously found that the addition of organic materials modulates soil bacterial community composition and abundance by elevating soil total nitrogen and organic matter levels (Yang et al., 2023). Moreover, the organic–inorganic fertilizer combination improves soil organic matter and microbial community structure more effectively than chemical fertilizer alone.

Research has shown that manure application significantly increases the alpha diversity of bacterial communities (Figure 3), which is consistent with previous findings on the addition of organic materials (Yang et al., 2023; Zhao et al., 2026). Bacterial diversity increased because manure/straw inputs supplied labile carbon that activated copiotrophic taxa (e.g., *Pseudomonadota* and *Bacteroidetes*), while manure-derived N and P alleviated stoichiometric constraints, broadening niche breadth and promoting species coexistence. Fungal diversity declined because manure shifted the soil toward a high-nutrient, low-lignin state, suppressing specialist lignocellulose-degraders (e.g., *Basidiomycota*, *Ascomycota*), while exogenous microbes intensified competition and eroded resident fungi. Furthermore, SOC, TN, MBN, and MBC were identified by this study as the main factors driving the increase in bacterial diversity (Figure 6). Studies have shown that soil bacteria are highly sensitive to environmental changes, and variations in soil carbon and nitrogen fractions influence microbial species composition (Yang et al., 2026). Manure application increases bacterial diversity by enhancing soil available nutrients (NO₃^−^-N, Olsen-P, and NH₄^+^-N) and decreasing soil pH (Figure 7). In summary, manure application boosts both dominant bacterial abundance and overall bacterial community diversity.

### 4.3 Under MNP, greater beneficial microbial abundance, and bacterial diversity improve soil quality and crop yields

It is widely recognized that microbial community structure and diversity play essential roles in maintaining nutrient cycling, organic matter decomposition, and crop yield (Zheng et al., 2019). Using a structural equation model, this study established relationships among soil properties, bacterial community composition and diversity, SQI, and crop yield (Figure 7a), revealing positive effects of both bacterial composition and diversity on the SQI. Notably, microbial diversity contributed more to the SQI than community composition. Manure is rich in stable organic matter and various nutrients, which buffer soil pH and increase soil available nutrients, thereby providing a favorable living environment for a wider range of microbial taxa (Guo et al., 2010). Higher microbial diversity implies greater functional redundancy and resistance to disturbance, enabling more stable maintenance of organic matter decomposition and nutrient cycling processes (Maron et al., 2018). Furthermore, bacterial diversity was positively influenced by soil environment and available nutrients. Consequently, manure application enhances soil conditions, promoting greater bacterial diversity and abundance of dominant microbes. These changes improve overall microbial community function, ultimately benefiting both soil quality and crop yield.

### 4.4 Outlook

Using a full-dataset approach in a long-term field experiment, this study evaluated treatment effects on soil quality and crop yield, identified the primary factors influencing soil quality, and elucidated how manure management affects soil quality via shifts in microbial community structure and diversity. These results provide valuable parameters for sustainable manure application and soil quality assessment. In the future, we plan to continue employing methods such as metagenomics and stable isotope probing to comprehensively elucidate the species composition, functional potential, and metabolic pathways of soil microbial communities, as well as their interactions with the environment and plants.

## 5 Conclusion

Our findings indicate that manure application (MNP and M) significantly improved SQI compared to conventional fertilization, with MNP being more effective than M. This improvement is largely due to enhanced soil physical, chemical, and biological properties, particularly SOC and TN. Under MNP, higher soil nutrient levels increased dominant bacterial abundance, while modified pH and available nutrients boosted bacterial diversity. Both shifts enhanced soil quality, with diversity playing a more effective role than community composition. In conclusion, long-term combined application of manure with mineral fertilizers improves soil quality, enhances bacterial diversity, and sustains crop productivity in rain-fed dryland agriculture.

## Data Availability

The datasets presented in this study can be found in online repositories. The names of the repository/repositories and accession number(s) can be found in the article/Supplementary material.

## Footnotes

1
https://cran.r-project.org
