# Supplementary material for: Long-term combined organic and inorganic fertilization enhances soil quality and crop yield by regulating bacterial diversity and soil physicochemical properties
Source: Front Microbiol. 2026 Aug 3;17:1909648. doi: 10.3389/fmicb.2026.1909648 (PMC13478087; doi:10.3389/fmicb.2026.1909648)
Supplement: Supplementary file 1 [file Data_Sheet_1.DOCX]

**Long-term combined organic and inorganic fertilization enhances soil quality and crop yield by regulating bacterial diversity and soil physicochemical properties**

Supplementary figure 1. Wheat-maize rotation system: Spring corn in 2022; Winter wheat from 2022 to 2025.

Supplementary figure 2. PCA of soil bacterial and fungal communities under the different fertilization management strategies. (a) PCA of soil bacteria. (b) PCA of soil fungi.

 Supplementary figure 3. Genus-level composition of soil bacterial and fungal communities under different fertilization management strategies. (a) Soil bacterial composition. (b) Soil fungal composition.

Supplementary figure 4. RDA of soil bacterial and fungal communities under the different fertilization management strategies. (a) RDA of soil bacteria. (b) RDA of soil fungi.
